# Analysis of User Interaction with a Brain-Computer Interface Based on Steady-State Visually Evoked Potentials: Case Study of a Game

**DOI:** 10.1155/2018/4920132

**Published:** 2018-04-15

**Authors:** Harlei Miguel de Arruda Leite, Sarah Negreiros de Carvalho, Thiago Bulhões da Silva Costa, Romis Attux, Heiko Horst Hornung, Dalton Soares Arantes

**Affiliations:** ^1^School of Electrical and Computer Engineering, University of Campinas (UNICAMP), Campinas, SP, Brazil; ^2^Institute of Exact and Applied Sciences, Federal University of Ouro Preto (UFOP), João Monlevade, MG, Brazil; ^3^Brazilian Institute of Neuroscience and Neurotechnology (BRAINN), CEPID-FAPESP, Campinas, SP, Brazil; ^4^Institute of Computing, University of Campinas (UNICAMP), Campinas, SP, Brazil

## Abstract

This paper presents a systematic analysis of a game controlled by a Brain-Computer Interface (BCI) based on Steady-State Visually Evoked Potentials (SSVEP). The objective is to understand BCI systems from the Human-Computer Interface (HCI) point of view, by observing how the users interact with the game and evaluating how the interface elements influence the system performance. The interactions of 30 volunteers with our computer game, named “Get Coins,” through a BCI based on SSVEP, have generated a database of brain signals and the corresponding responses to a questionnaire about various perceptual parameters, such as visual stimulation, acoustic feedback, background music, visual contrast, and visual fatigue. Each one of the volunteers played one match using the keyboard and four matches using the BCI, for comparison. In all matches using the BCI, the volunteers achieved the goals of the game. Eight of them achieved a perfect score in at least one of the four matches, showing the feasibility of the direct communication between the brain and the computer. Despite this successful experiment, adaptations and improvements should be implemented to make this innovative technology accessible to the end user.

## 1. Introduction

A Brain-Computer Interface (BCI) is a system able to directly associate the brain activity to a command to be operated by a computer or an electrical device, bypassing the output pathways (nerves and muscles) of a standard device of interface, which makes it attractive for the development of assistive technologies, such as automatic wheelchairs [1, 2], robotic arms [3], and speller communication [4], as well as for entertainment applications, such as games, augmented reality, and virtual reality [5–8].

One of the first BCIs was developed in 1964 by Dr. Grey Walter. During a surgery for another reason, Dr. Walter placed electrodes on the motor cortex of a patient and recorded the brain activity while the patient pushed a button to advance a slide projector. Subsequently, the system was connected to a projector and allowed the patient to advance the slides even before he/she had actually pushed the button [9]. Since then, BCI systems have been the focus of many researches that have contributed to the advancement of technology and understanding of the human brain [10].

Interface devices that mediate the interaction between humans and computers should be as simple, secure, precise, and enjoyable as possible. The research field of Human-Computer Interface (HCI) aims precisely at the development of such interfaces, so that the user experience occurs in the best possible way. However, in the context of BCI systems, the guidelines are not yet consolidated.

The present study analyzes a BCI system based on Steady-State Visually Evoked Potentials (BCI-SSVEP) from the perspective of HCI, in such a way as to understand how the elements of the interface affect the user and how the interaction occurs. For this purpose, a game with four commands controlled by BCI-SSVEP has been developed and tested in a controlled experiment involving 30 volunteers.

Results include a large database of brain signals linked to the users' perception about various aspects of the graphical user interface and the interaction with the application. Qualitative and quantitative considerations about acoustic feedback; shape, position, and contrast of visual stimuli; visual fatigue; background music; feeling of control; among others, are presented and discussed. The whole experiment and observations constitute a rich and important material to assist in future projects on BCI systems, especially for BCI-SSVEP with visual stimulation projected on a screen.

### 1.1. BCI Based on SSVEP

**Figure 1:**
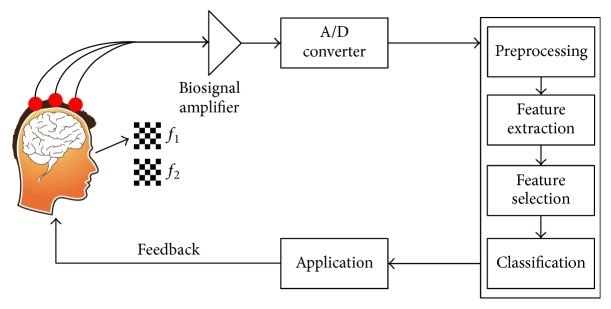
Diagram of a BCI based on SSVEP system.

A BCI is a closed-loop system that acquires and analyzes brain signals, in such a way as to establish a communication channel between the brain and an application, as shown in Figure 1. The development of a BCI requires multidisciplinary skills, involving knowledge about functional aspects of the human brain, computer systems, and engineering. The system can be modularized as follows: (1) acquisition of brain activity, (2) processing of brain signals, and (3) generation of the commands to be executed by an application. In turn, the application performs some actions perceived by the user, constituting the system feedback [11].

A BCI system can be classified as exogenous or endogenous, depending on the nature of the recorded signal. Exogenous BCI systems depend on neuron activity evoked by external stimuli. In contrast, endogenous systems do not rely on external stimulus, since they are based mainly on brain rhythms and other potentials. In this article, the focus is on exogenous BCI-SSVEP [12]. The SSVEP is a neurophysiological response to a visual stimulation. When a user is visually stimulated by a LED, lamp, or an image projected on a screen that flickers at a well-defined frequency, the electroencephalographic records from his/her occipital lobe are synchronized with the frequency of the stimulus. Therefore, the analysis of the brain signal allows to identify the frequency of the stimulus to which the user was exposed. A BCI-SSVEP employs several visual stimuli, each one flickering at a different frequency and associated with a command of the application [13].

In the present study, the BCI-SSVEP developed by our research group in the School of Electrical and Computer Engineering at the University of Campinas was used to control our game, called “Get Coins” [14, 15]. The details of each module of our BCI system are described in the following.

## 2. Materials and Methods

### 2.1. Acquisition of Brain Signals

The acquisition of a brain signal can be invasive, in which case the electrodes are placed on the cortex by surgical procedures, or noninvasive, a case not requiring a brain surgery. The electroencephalography (EEG) procedure is a usual noninvasive technique employed to measure brain activity. In this approach, the electrodes are positioned directly on the scalp [9] and the EEG records present a signal-to-noise ratio (SNR) lower than that obtained with invasive techniques.

In the present study, the EEG was employed, since it does not expose the volunteers to the risks of a surgery, is cheaper, and allows easy, fast, and safe assembling of the electrodes. The equipment used for brain signal recording were the g.SAHARAsys® with 16 dry-electrode and the g.USBamp® biosignal amplifier [16]. The signal was recorded at a sample rate of 256 Hz using MATLAB®. Before starting signal acquisition, the following procedures were performed: channel calibration; verification of the electrode impedance calibration (not exceeding 5.0 kΩ); connection of the ground and reference on mastoids; and waiting for the stabilization of the signal. The electrodes were arranged at O1, O2, Oz, POz, Pz, PO4, PO3, PO8, PO7, P2, P1, Cz, C1, C2, CPz, and FCz, according to the international 10-10 system [17].

**Figure 2:**
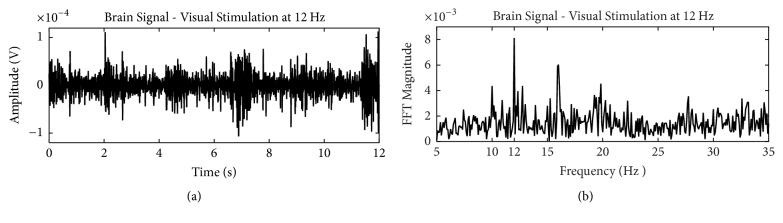
EEG signal with SSVEP response for a stimulus flickering at 12 Hz: (a) time domain and (b) frequency domain.

Figure 2 shows an example of EEG signal recorded on the visual cortex (Oz position) when a user was exposed during 12 seconds to a stimulus flickering at 12 Hz.  Figure 2(a) shows the signal in the time domain and Figure 2(b) the spectrum of the signal from which a peak at 12 Hz can be identified.

### 2.2. Brain Signal Processing

The signal processing can be divided into four stages: preprocessing, feature extraction, feature selection, and classification. The purpose of the preprocessing is to improve signal quality by increasing the SNR. The feature extraction consists of describing the information embedded in the brain signal succinctly. The feature selection realizes a filtering of the most relevant features necessary to discriminate the classes (stimuli/commands). Finally, the classifier interprets the brain signal through the features and generates the control signal for the application.

In the following subsections, we describe how each stage was designed for this study.

#### 2.2.1. Preprocessing

To remove the smooth displacement and electromagnetic artifacts, the EEG signal was filtered by an analog Butterworth bandpass filter (5–60 Hz) of order 8 and by a notch filter (58–62 Hz) of order 4. To remove other artifacts, as eye blinking, the data were then submitted to a spatial filtering using the Common Average Reference (CAR) method, defined as

(1) $$\begin{array}{c} V_{i}^{CAR}=V_{i}^{ER}-\frac{1}{n}\overset{n}{\underset{j=1}{\sum }}V_{j}^{ER}, \end{array}$$

in which *V*_*i*_^ER^ is the potential of the *i*th electrode measurement with respect to a common reference, and *n* is the number of electrodes in the array, in our case *n* = 16. The average value is subtracted from the potential of each electrode, eliminating artifacts present in most them. As simple as it may be, CAR is an effective solution to improve the SNR and the BCI-SSVEP performance [15].

#### 2.2.2. Feature Extraction

The stage of feature extraction is responsible for representing the input data in a compact way, reducing their dimensionality. The process is conducted without loss of the information that allows to discriminate the stimuli. Indeed, the feature extraction should emphasize the relevant characteristics of the input signal to facilitate the task of the classifier.

For EEG signals with SSVEP response, a classical feature is the spectral amplitude estimated by the Fast Fourier Transform (FFT) algorithm. In the present case, every two seconds 512 brain signal samples were recorded on channel *i*, generating the following features subvector *A*_ch_*i*__ with four inputs, corresponding to the peak values of the FFT at frequencies 6, 10, 12, and 15 Hz:

(2) $$\begin{array}{c} A_{{ch}_{i}}=\left[\begin{array}{cccc} a_{6\;Hz,{ch}_{i}} & a_{10\;Hz,{ch}_{i}} & a_{12\;Hz,{ch}_{i}} & a_{15\;Hz,{ch}_{i}} \end{array}\right]. \end{array}$$

The following features vector *H*, with 64 entries, stores the four features, for the 16 electrodes, every two seconds of brain signal recording:

(3) $$\begin{array}{c} H=\left[\begin{array}{cccc} A_{{ch}_{1}} & A_{{ch}_{2}} & \cdots & A_{{ch}_{16}} \end{array}\right]. \end{array}$$

#### 2.2.3. Feature Selection

Part of the features in vector *H* can be eliminated to further reducing the dimensionality of the problem. The purpose of feature selection is to use just the data that provide useful information to discriminate the classes, eliminating redundant information and those that may impair classifier performance.

Feature selection can be performed with filter or wrapper techniques [18, 19]. The filter approach uses statistical measures to quantify the relevance of each feature, whereas the wrapper approach ranks the characteristics according to the classifier performance. For the feature selection problem in BCI-SSVEP systems, the search in the feature space using greedy heuristics, called forward wrappers, has been shown to be quite efficient [15]. This technique considers the set of features used in the training step together with the classifier to select the set of features that provides the best performance for the BCI system. The algorithm used here works as follows:

- Initially, the BCI performance for each subvector *A*_ch_*i*__ alone is evaluated; that is, the data coming from each electrode are tested one by one, individually.
- Subsequently, the subvector *A*_ch_*i*__ that provides the best accuracy is maintained, and the system performance is evaluated by combining *A*_ch_*i*__ with *A*_ch_*j*__, for *i* ≠ *j*.
- The progressive inclusion of new *A*_ch_ ‘s continues as long as the system performance increases. The stopping criteria were as follows: (1) when performance degradation occurs for two consecutive times with any new combination; (2) when the signals coming from all 16 electrodes are already employed.

After applying the forward wrappers algorithm, the feature vector *H* is reduced, resulting in a vector $\tilde{H}$ of order less than or equal to 64.

#### 2.2.4. Classification

The last stage of the signal processing module is the classification. The classifier must evaluate the characteristics of the vector $\tilde{H}$ and identify the stimulus to which those features correspond.

A linear classifier based on the least squares method was used. This approach is computationally inexpensive and is a well-established technique in the literature for discriminating signals with SSVEP response [15].

The classifier comprises two steps: training and operation. In the first step, the system is fed with the labeled features of the four classes and the separation hyperplanes are generated by solving the following equation:

(4) $$\begin{array}{c} \omega_{c}={\left(\;X^{T}X\;\right)}^{-1}X^{T}r_{c}, \end{array}$$

in which *X* is the feature matrix, composed of several vectors $\tilde{H}$, *X*^*T*^ is the transpose of *X*, and **r**_*c*_ is the vector of labels of class *c*, with entries +1 for the corresponding class and −1 for the other classes. In our study, *X* has 192 entries, being 48 for each class (stimulus).

In the operation step, the user is controlling the application at run time. The output of the classifier is given by solving the following expression for each hyperplane:

(5) $$\begin{array}{c} y_{c}=\hat{H}\omega_{c}, \end{array}$$

with

(6) $$\begin{array}{c} \hat{H}=\left[\;\tilde{H};1\;\right]. \end{array}$$

Ideally, the variable *y*_*c*_ must have a +1 if it belongs to the class *c*, and −1 otherwise. As a decision criterion, if more than one solution *y*_*c*_ presents positive values, it is decided as the class with the highest value of *y*_*c*_ [20].

### 2.3. Application: The Game “Get Coins”

**Figure 3:**
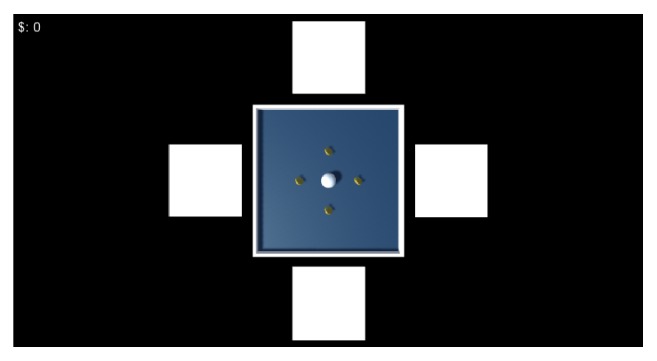
Screenshot of the “Get Coins” game.

We have developed a computer game, here called “Get Coins,” using the Unity3D® game engine, to evaluate the user interaction with an application controlled by the previously presented BCI-SSVEP.  Figure 3 shows the game screen. The main goal of this game is to collect as many coins as possible by moving the small ball around the board. The simplicity of the game makes its objective and mechanisms quite intuitive, allowing an easy understanding for people with different familiarities with computer games and thus minimizing the influence of game characteristics on the study's objective, which is to evaluate user interaction with an BCI-SSVEP.

The direction of the small ball is determined by the four stimuli positioned intuitively on the sides of the board corresponding with the commands to move the small ball to the left, right, down, and up. The stimuli are squares that alternate between black and white in the frequencies of 6 Hz (left), 10 Hz (right), 12 Hz (down), and 15 Hz (up). The players can give a command every two seconds, during which time they should gaze at the stimulus corresponding to the desired command. The period of two seconds was chosen considering the compromise between the system hit rate and the user's visual fatigue. A long time of concentration in the stimulus leads to a more intense SSVEP response in the spectral analysis of the signal, which contributes to a better performance of the system. On the other hand, very long periods lead to visual fatigue, stressing the user and compromising the dynamics of the game.

When the player collects a coin, the counter located on the upper left side of the screen is incremented by 1. The player has two minutes, corresponding to 60 movements, to collect the four coins. The game is ended after the player has collected all coins or after two minutes.

**Figure 4:**
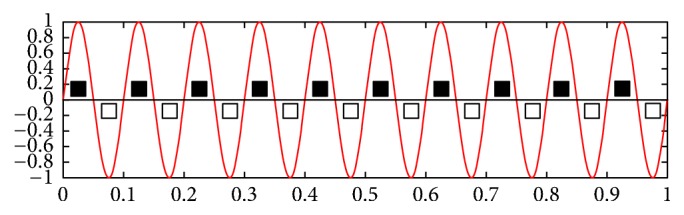
Stimulus at 10 Hz generated by a sine wave.

A key point in the development of the interface for BCI-SSVEP is to guarantee precision in the flickering rate of stimuli [13]. In the present study, a sine wave has been generated internally to change the visual stimuli from black to white and vice versa, in well-defined frequencies.  Figure 4 shows a 10 Hz sine wave in an interval of 1 s, alternating the pattern of the stimulus at each zero-crossing of the sine wave, generating the flickering stimulus in the desired frequency of 10 Hz.

Also, two feedback modalities were included: visual and acoustic. The visual feedback is given by the movement of the ball, while the acoustic feedback consists of a beep sounded after each movement. The beep informs that a command was executed, avoiding that the user loses concentration on the stimulus to visualize the movement of the ball. During the game, a log file is generated by fetching the time spent to collect the coins, the number of steps taken by the ball and the path traveled by the ball.

Before arriving at the final version of the game presented, an inspection was conducted by four HCI experts from the Institute of Computing at the University of Campinas. The ten usability heuristics for user interface design, proposed by Nielsen, were used to evaluate the game interface [21]: (1) visibility of system status; (2) match between system and the real world; (3) user control and freedom; (4) consistency and standards; (5) error prevention; (6) recognition rather than recall; (7) flexibility and efficiency of use; (8) aesthetic and minimalist design; (9) helping users recognize, diagnose, and recover from errors; and (10) help and documentation. The main recommendations were as follows:

1. Adjust the position of the coins in such a way as to require a number of steps to collect them compatible with the time that the players have to complete the game.
2. Limit the duration of the game in 120 seconds to avoid fatigue of the player.
3. Increase the size of the small ball to allow its visualization through peripheral vision.
4. Insert a coin counter at the top to guide and motivate the players about their performance.

### 2.4. Experimental Setting

A total of 30 volunteers aged from 20 to 45 years, average 29.93 ± 6.11, being 22 males and 8 females, have participated in this study. Half of the volunteers reported to play digital games frequently and the other 15 stated that they had not played any digital game before. All of them were adequately informed about the research and the experimental protocol and signed the consent form approved by the Ethics Committee of the University of Campinas (n. 791/2010). All volunteers were healthy individuals, with normal or corrected for normal vision.

**Figure 5:**
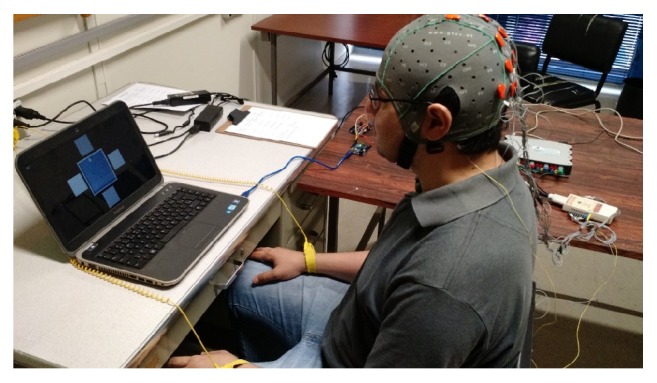
Experimental setup.

The experiment was performed in a room with low light intensity to avoid interference from lightning. The volunteers were seated at approximately 70 cm from the monitor and were instructed to remain as motionless as possible to avoid mechanical artifacts. They made use of an antistatic wrist strap to discharge electrostatic energy. The cap with 16 dry electrodes was positioned on the scalp, as shown in Figure 5 with the experimental setup.

**Figure 6:**
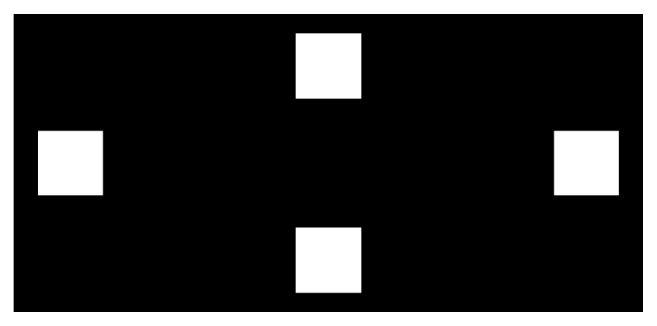
Training screen.

The experimental protocol consisted of training, playing, and answering a personal perception questionnaire. During the training, a screen with four stimuli, as shown in Figure 6, was presented. The visual stimulation setup followed the same standards during training and online procedures. The volunteers were informed about the need of focusing their gaze on specific visual stimulus by 12 seconds. The stimulus to be focused and the initial and final time were informed orally. The process was repeated eight times for each of the four stimuli. The recorded brain signal was used to train the classifier of the BCI and to estimate the expected performance of the player.

After the training, the game “Get Coins” was introduced to the volunteer along with a tutorial on how to play. The volunteers played five versions of the game, each one evaluating different aspects of interface and interaction, as shown in Table 1.

All versions of the game were played in a random order for each volunteer, in such a way as to minimize the bias of the results due to fatigue or learning of the player. In Version 2, the game was controlled by the keyboard, to compare this input device with the interaction via BCI.

At the end of each match, volunteers answered a questionnaire with continuous scale items about their perception. The questions and the ranges are presented in Table 2.

Moreover, the following assertive questions with yes/no answers were asked:

- Did you feel your eyes watering?
- Did you feel dizzy?
- Did you think about quitting in the middle of the game?
- Did you feel uncomfortable posture?

The questionnaire additionally had an optional field for comments and suggestions.

This qualitative information together with the quantitative data recorded in the log file (collecting the course of the ball, number of steps, total time of play, and number of coin catches) has allowed us to draw a parallel between the perception of users and their performance in the game. All data were statistically evaluated and the *p* value was estimated using the Wilcoxon *t*-test for the comparison of two groups and the ANOVA model for the comparison of three or more groups. The confidence value was set at 95%.

## 3. Results and Discussion

The experiment allowed the generation of a brain signal database from 30 individuals collected during the training stage, containing 8 repetitions of 12 s for the four frequencies (6, 10, 12, and 15 Hz). Additionally, the users' perception about the interface elements and their interaction with the game were registered. All these data have significantly supported the present study for a better understanding on how interaction with BCI-SSVEP occurs.

All the 30 volunteers performed the entire experimental procedure, that is, training, playing of the five matches, and answering the questionnaire. None of the volunteers have asked to interrupt the experiment, indicating that eventual distresses caused by the electrode cap, visual stimulation, or fatigue were tolerable. The average duration of an experimental session was 34′38′′ ± 04′51′′.

**Figure 7:**
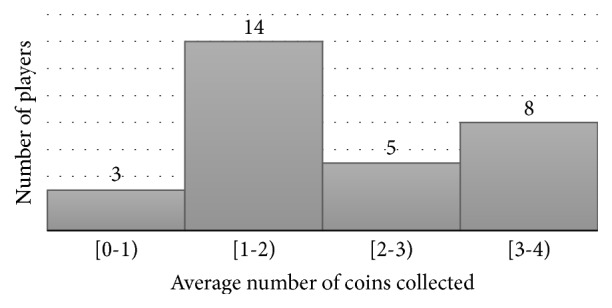
Histogram of average number of collected coins.

Despite equal conditions, the hit rate was different for each volunteer, as expected, since the BCI system depends on the neurophysiological response and biological and cognitive factors of the individuals, as well as on their concentration on stimulus and abilities. Eight volunteers collected all the coins in at least one of the versions of the game using BCI. Four of them collected all coins in all game versions. Although the game is time-limited to 120 seconds, these four individuals needed an average time of 76.94 ± 16.36 seconds to collect all coins. On the other hand, four other volunteers did not collect any coins in exactly one of the game versions, and one volunteer did not collect any coins in two game versions.  Figure 7 presents the average number of collected coins, considering just the game versions controlled by BCI.

Considering the five versions of the game separately, the number of collected coins is shown in Table 3. Only in Version 2, controlled by keyboard, all the volunteers collected all the four coins. A statistically significant difference of the average of collected coins was detected only between Version 2 and each of the other versions (*p* < 0.0001). Furthermore, considering only the versions controlled by BCI, the average number of coins collected was 2.05 ± 1.26, and it remained constant throughout the game, indicating that the fatigue and learning factors did not quantitatively influence the performance of volunteers.

**Figure 8:**
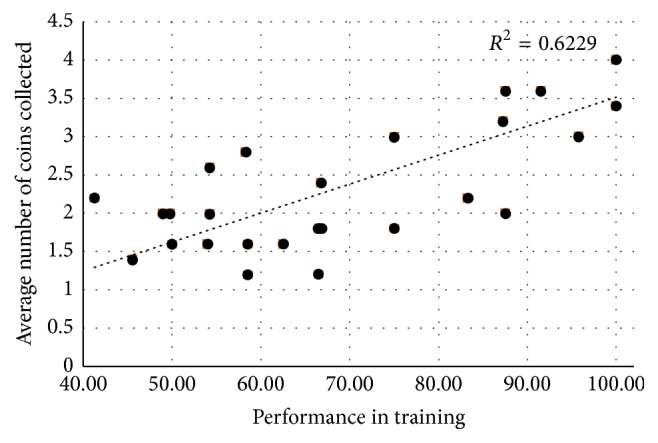
Relationship between the expected performance using the training data and the average number of coins collected.

Another important point is that the predicted performance using the training data did not always correspond directly to the performance achieved during the online application, as shown in Figure 8. Despite a trend in direct correspondence between the two performances, some users with high performance in the training session presented poor performance in the game and vice versa. Some of the reasons that may explain this behavior are as follows: in the online version, the volunteer is motivated and has a well-defined goal; however there is movement of the eyes for transitions between commands and visual stimuli and distraction between the stimuli and the game board. However, these factors act differently for each volunteer.

Regarding the motivation to perform the training stage, the volunteers indicated that they felt motivated with an average of 6.98 ± 1.98, regarding a maximum of 10 for “very motivated.” During the training stage, two volunteers reported fatigue and one related having experienced involuntary spasms in the eyes. In fact, the training stage was really a “tiring stage,” requiring a concentration of eight times 12 seconds on each of the four visual stimuli. A possibility of reducing this fatigue would be to decrease the number of samples to train the system; however this could degrade the system performance. To ensure a better hit rate, and consequently greater controllability of the game, we had decided to keep the eight repetitions in the training stage.

About the perception of fatigue caused by the game, there is a statistically significant difference only between Version 2 of the game and the other versions (*p* < 0.0001). The average values are presented in Table 4 (with 0 being very tiring–10 very invigorating). Thus, we can conclude that the control via BCI is more tiring than via keyboard, but the fatigue is acceptable (average of 5.59 ± 1.83). A quantitative analysis indicates that the users need to execute almost twice as many commands to complete the goals of the game using the BCI (average of 49.45 ± 11.29) compared to using the keyboard (average of 24.60 ± 2.43).

The perceived distress or comfort caused by the visual stimuli was neutral (an average of 5.84  ±  1.78), that is, neither very comfortable (10) nor very uncomfortable (0). The distress did not change statistically during the sessions, considering the beginning and the end of the experiment (*p* = 0.6550).

According to the perception of users, the distress/comfort caused by the cap with electrodes was 6.94 ± 2.01, with 10 being very comfortable and 0 very uncomfortable. Considering the markings performed at the beginning and at the end of the experiment, by each volunteer, this remains constant, and there is no significant difference (*p* = 0.5826). This indicates that users are likely to accept the regular use of the cap and electrodes on scalp. However, the EEG acquisition system would need to be improved for frequent use, since the correct positioning of the electrodes is not trivial for an ordinary user. Also, for an actual application, it is unreasonable to require the user not to move the head. However, this movement can displace the electrodes or even cause the loss of contact with the scalp, seriously compromising the BCI performance. There already exist some solutions like EMOTIV Epoc+ [22] that have prepositioned, fixed electrodes, and the data transmission of the electrodes is via a wireless channel, which allows free movement of the head.

The well-defined goal of the game also served as a motivation, possibly distracting from or reducing the fatigue and the distress caused by the cap and by visual stimuli. In fact, some applications may require longer interactions, so that minimizing visual distress and fatigue should be a central requirement in designing interfaces for applications controlled by BCI-SSVEP. Indeed, the fatigue can lead to loss of concentration, which can compromise the intensity of the SSVEP response and, consequently, the performance of the system [23].

Regarding the sense of dominion of game controls, the players indicated to exercise a medium to low control using BCI, averaging 5.61 ± 2.73 (0 being total control–10 no control), against an almost total control with keyboard, averaging 0.91 ± 2.53 (*p* < 0.0001). However, they indicated a neutral position regarding the fun of the keyboard game control, compared to an average of 4.99 ± 3.72 (0 being boring–10 fun) regarding the BCI versions. In the field for comments and suggestions, nine volunteers had reported difficulty in moving the ball to the desired direction. The BCI system sometimes leads to classification errors and ends up executing a command that does not correspond to the one desired by the user, leaving the player with a sense of little control over the game. However, contradictorily, one among these nine volunteers achieved total success in all the game versions, collecting all the four coins. In other words, he presented an excellent control although in his perception he felt without control of the game. The keyboard version was controlled with the directional arrow keys, and the players indicated this as intuitive, with an average of 8.98 ± 2.05. For the BCI versions, they also indicated that the placement of the stimuli on the monitor made the commands intuitive, with an average of 7.65 ± 2.49 (10 for very intuitive).

In general, the players liked the game in both modes of control, with an average of 7.09 ± 2.10, for BCI and 7.04 ± 2.01 for keyboard (10 for pleasant). As for the challenge of the game, the players indicated that the game via the keyboard is very easy, with an average value 0.88 ± 2.04, and that the game is more challenging when the control is via BCI, with an average of 6.01 ± 3.31 (0 very easy–10 very challenging; *p* < 0.0001).

**Figure 9:**
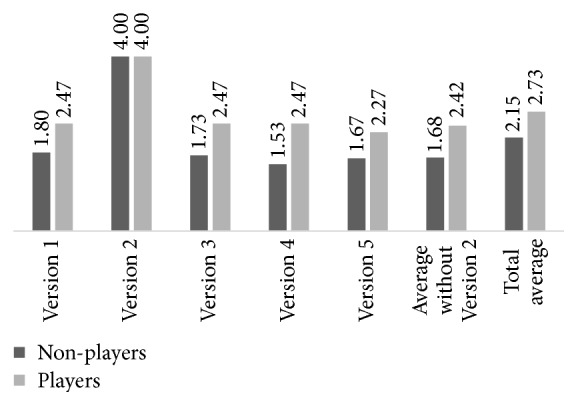
Average number of collected coins in each version of the game for players and nonplayers volunteers.

As far as acoustic feedback is concerned, the volunteers reported that it assists in game control, with a statistically significant difference (*p* = 0.0426) between the versions of the game with acoustic feedback (1, 3, and 5) and the Version 4 without acoustic feedback. However, the performance in terms of the number of collected coins was not statistically different (*p* = 0.3810). Although the performance in the game was not statistically different, acoustic feedback is important for the player to know what is happening in the game without losing the focus on the visual stimulus, mainly for volunteers who had no experience with computer games (see Figure 9). Also, in the comments/suggestions field of the questionnaire, two volunteers suggested that different beeps for each direction of the ball could better assist in feedback. These observations reiterate the importance of acoustic feedback.

Still in relation to sound effects, the amount of collected coins was not significantly different (*p* = 0.7188) between Version 3, with background music, and the other versions of the game without background music. In the perception questionnaire, the volunteers reported that background music was almost irrelevant (average of 3.37 ± 2.98, 0 being irrelevant), but it does not disturb either (average of 6.77 ± 3.33, 10 being no disturbances). This result is especially interesting, because it is impossible to control the noise level in a generic environment. Results indicate that background sounds tend not to impact considerably on the performance of individuals, either quantitatively or qualitatively. However, in the present study the background music was part of the context of the application, so further investigation is necessary to check the impact of random sounds, such as people talking, traffic, and sudden sounds.

Regarding the background color, we verified that, according to the perception of the users, both backgrounds, black and gray, were pleasant with averages of 6.81 ± 1.94 for black and 6.26 ± 2.25 for gray (10 for very pleasant), and there was no statistically significant difference between the perception of the users in the two cases (*p* = 0.3837). Considering the amount of coins collected, for the black background, the average number of collected coins was 2.13 ± 1.22, while for the gray background the average was 1.97 ± 1.19. Although Version 5 of the game with gray background and a lower contrast has shown a smaller average of collected coins, there was no statistically significant difference between the performances (*p* = 0.3629).

The 15 volunteers who affirmed to play computer games performed slightly better than the other 15 who reported not to play. This was verified for all the versions of the game controlled by BCI (Figure 9). However, there is no statistically significant difference on the average performance between the two groups at a 95% confidence level (*p* = 0.0529). Possibly, this better performance of the group of players is because they are accustomed to focus on the screen during a game and well-acquainted at developing mental strategies to achieve the goals.

**Figure 10:**
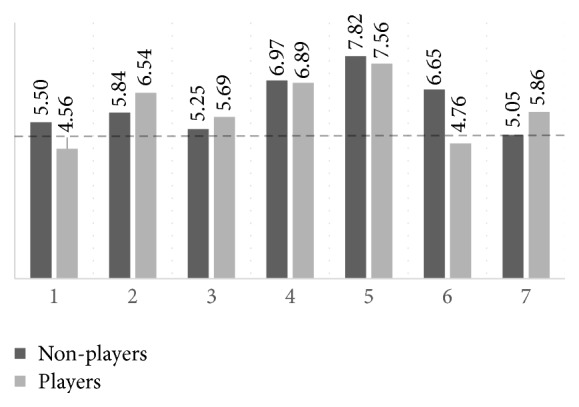
Comparison between the perception of players and nonplayers volunteers related in Version 4 of the game.

Considering Version 4 of the game (no acoustic feedback and no background music), which presented the greatest discrepancy in the average between the two groups, Figure 10 shows the perception of the volunteers regarding the following parameters:

1. Fatigue caused by the game: 0 very tiring–10 very invigorating
2. Visual comfort of the stimulus: 0 very uncomfortable–10 very comfortable
3. Acoustic feedback helps: 0 not at all–10 a lot
4. Game challenge: 0 very easy–10 very challenging
5. Intuitiveness of game controls: 0 not intuitive at all–10 very intuitive
6. Control over the game controls: 0 total control–10 no control
7. Fatigue caused by the game: 0 very tiring–10 very invigorating.

There is no remarkable difference among the average values between the group of players and nonplayers (*p* > 0.05). The greatest difference between averages is observed at column 6 of Figure 10, about sense of control. Although the volunteers of the group of players performed better, they paradoxically reported having a lower perception of control of the game (4.76 ± 2.07) than the group of nonplayers (6.55 ± 2.85), but without statistical significance (*p* = 0.0529). This is probably because the volunteers accustomed to play tend to have a more effective sense of control of game commands using classic interaction devices, as keyboard, mouse, or joystick. This also impacted on the greater sensation of fatigue reported by the group of players for both training (column 1 of Figure 10) and playing (column 7 of Figure 10).

In relation to gender of volunteers, 8 were women (of these, 6 were nonplayers) versus 22 men, being 9 nonplayers. The average of collected coins, considering all versions controlled by BCI, was 1.81 ± 1.26 for women and 2.14  ±  1.13 for men, without statistically significant difference (*p* = 0.4513) between the performances.

Despite the great potentiality of the BCI system, as we have confirmed here, especially in assistive applications in which this tool may be the only viable way to control a device, the information transfer rate is still much smaller than those provided by conventional input means, such as the keyboard [9].

In the field for comments and suggestions of the questionnaire, some volunteers highlighted some contradictory opinions. For example, a volunteer reported that the interface with gray background was more enjoyable and less tiring than the black background. Another volunteer reported exactly the opposite. This indicates that the interface should be, as far as possible, customizable to suit the preferences of each user.

## 4. Conclusions

The possibility of using BCIs to control a device without need of nerves and muscles makes this technology quite promising, specially to conceive assistive technologies and entertainment applications. Despite the potential of this technology and the encouraging results already achieved in the scientific community, BCI systems are still at the developmental stage.

In the present study, 30 volunteers played the “Get Coins” game. The results have allowed to test several characteristics of the interface, as well as to analyze the user interaction using a BCI-SSVEP and to compare system performance and user interaction with a classic control device, such as the keyboard.

None of the volunteers had prior experience in controlling games by BCI. All volunteers understood the goals of the game and played five matches, four using the control via BCI and one using the control via keyboard. All volunteers collected at least one coin in the matches controlled by BCI, while four collected all the coins in all game versions. The total average of the number of collected coins indicates the feasibility of this technology to control an application. When the game was controlled by keyboard, all volunteers collected the four coins. Familiarity with the keyboard, with its high accuracy and precision, and the simple goals of the game offered a very low challenge in this mode of control. This indicates that game concept and mechanism did not influence our experimental results.

Regarding the fatigue caused by the game, volunteers reported that the version of the game controlled by the keyboard was less tiring than by BCI, which is understandable since the matches with keyboard were faster than matches with control via BCI. Also, the control by keyboard does not require concentration on stimuli. However, the BCIs may be the only option for people with reduced mobility, and it is interesting to note that it is a valid option despite its current limitations.

About the characteristics of the interface, the volunteers reported that acoustic feedback helped control, since it indicates that a command has been executed. However, the performance in terms of the number of collected coins was not statistically significant. As for the background music, users indicated that neither its presence nor its absence influenced the game play and should therefore be an element to be optionally offered to each player. This also indicates that background noise, at reasonable levels, tends to be irrelevant and does not disturb concentration. The black and gray background intensity did not result in perceptual visual fatigue due to higher or lower contrast, nor did it affect the performance of users. Although three volunteers reported visual distress at some time during the experiment, they all decided to continue the experiment to the end. The distress felt by the volunteers at the beginning and the end of the experiment were not statistically different, probably because the matches were only 2 minutes long and possibly due to the novelty factor. Since none of the volunteers had had experience in controlling a game by brain signals before, this could also have minimized the sensation of fatigue caused by the visual stimuli.

As for the distress caused by the electrode cap, the level was not significant and remained constant throughout the experiment, showing that the volunteers did not bother with this in the experimental context.

Of the 30 volunteers who participated in this experiment, 15 had not played any type of digital game and 15 had played. Comparing the performance between these two groups, we observed that there was no significant statistical difference in performance between them. However, the group of players performed better than the nonplayers in all game versions, possible because of the concentration skills acquired through game playing. Further studies, however, are needed to understand this relationship.

The results of our study did not consider the impact of the learning effect on the interaction of users with BCI-SSVEP systems, as each volunteer took part in a single experimental session. Moreover, only healthy volunteers participated in the experiment, assessments with patients with motor, visual, mental, and hearing impairment should be better evaluated in future studies.

This research directs developers to understand users' difficulties and how the interaction of the user with a BCI based on SSVEP occurs. Additional research should aim at understanding more about this, in order to achieve more complete guidelines on how BCI applications should be constructed. Different from other interaction devices as mouse, keyboard, and joystick, BCI systems depend on the user's ability to concentrate on visual stimuli, so the interfaces must be designed to avoid distraction and fatigue. In fact, the study of BCI systems from the HCI point of the view is essential to understand the real needs of the individuals and to overcome the challenges to make BCI systems a reality for the end user.

## Figures and Tables

**Table 1:** Versions of the game Get Coins.

	Visual stimulus	Acoustic feedback	Background scenario	Background music	Control by BCI	Control by keyboard
Version 1	Yes	Yes	Black	No	Yes	No
Version 2	No	Yes		No	No	Yes
Version 3	Yes	Yes		Yes	Yes	No
Version 4	Yes	No		No	Yes	No
Version 5	Yes	Yes	Gray (50%)	No	Yes	No

**Table 2:** Questionnaire items, translated from Brazilian Portuguese.

Question topic	Range limits
Cap comfort	0 very uncomfortable–10 very comfortable
Visual comfort of the stimulus	0 very uncomfortable–10 very comfortable
Fatigue caused by training/by the game	0 very tiring–10 very invigorating
Motivation for training	0 very boring–10 very exciting
Game challenge with BCI/keyboard	0 very easy–10 very challenging
Background color	0 unpleasant–10 pleasant
Pleasantness of the positioning of the stimuli	0 very uncomfortable–10 very comfortable
Pleasantness of background color	0 very uncomfortable–10 very comfortable
Acoustic feedback helps	0 not at all–10 a lot
Background music disturbs	0 not at all–10 a lot
Background music lacks	0 not at all–10 a lot
Intuitiveness of game controls	0 not intuitive at all–10 very intuitive
Dominion of game controls	0 total control–10 no control
Control by keyboard is	0 boring–10 fun
Pleasantness of game	0 unpleasant–10 pleasant

**Table 3:** Average number of coins collected in each version of the game.

Version	Collected coins
1	2.13 ± 1.22
2	4.00 ± 0.00
3	2.10 ± 1.30
4	2.00 ± 1.39
5	1.97 ± 1.19

**Table 4:** Average fatigue caused by the game.

Version	Fatigue in the game
1	5.58 ± 1.78
2	7.51 ± 2.19
3	5.63 ± 1.83
4	5.46 ± 1.97
5	5.70 ± 1.79
